# Case of Obstinate Gleet

**Published:** 1807-05

**Authors:** 


### *To the Editors of the Medical and Physical Journal.*

Gentlemen,

I Shall feel myself particularly obliged by your inserting the following case in your next Journal; and if any of your readers conceive that the employment of different, or persisting in the same means, will in the end perfect a cure, will thank them to mention it. The case at present is, in my opinion, no other than an obstinate Gleet, but I may be mistaken; however, I leave it to the medical world to determine.

I am, &c.

*March* 15, 1807. H——. S——.

---

About six months ago, I was requested by a gentleman to visit a lady of his acquaintance, whom I found affected with the usual symptoms of incipient fever, added to which, she complained of a swelling in the right groin, difficult and painful micturition, with a sense of tension and heat about the vagina and adjacent parts, but she as yet, had not perceived any discharge. These symptoms first made their appearance the day before I was called, and proceeded, as she then imagined, from fatigue, and the non-appearance of the menstrual discharge; but it was clear to me, they had a venereal origin.

I immediately gave her an infusion of senna with cathartic salt, ordered her to drink plentifully of diluting liquids, and to abstain from exercise. The cathartic having operated very sufficiently, she took at bed-time pulv. ant. Lond. gr. vj. The following morning she was much better, the febrile symptoms were abated, and menstruation had commenced; but the same heat, tension, and difficulty of making water continued; the tumour in the groin was more painful, and not diminished in size. Capt. sal. nitri pulv. g. arab. aa gr. x. decoct. hord. ℥ij. 4. q. h. the diluting drinks to be continued. In about four days the pain had nearly ceased, an immense discharge of purulent matter took place from the vagina, and the swelling in the groin was much increased in size, but she assured me no sore, or abrasion of the cuticle, was to be perceived. Immediately ordered ungt. hydr. fort. ʒj. to be rubbed in at bed-time, gave her a saturnine lotion, and a mercurial plaster for the swelling in the groin, and ordered the nitrous medicines to be continued; by persisting in these means, the discharge abated, the pain and swelling disappeared, but numerous pustules, occasioning great itching and painful heat, broke out upon the labia and parts adjoining. I then substituted a solution of zinc. vitriol. (in the proportion of gr. jß. to ℥j. aquæ) for the saturnine wash, and having kept her mouth sore for about a week, after the disappearance of the tumour, gradually left off the mercury; the nitrous medicine occasioning nausea was likewise likewise discontinued; she shortly after perceived great amendment; the discharge was trifling, and the pimples always disappeared on applying the lotion. I now flattered myself the cure would be speedily compleated, but am disappointed; in spite of all my endeavours, she still continues in the same state; indeed, the discharge sometimes disappears for a day or two, but returns in increased quantity, and the eruption has never entirely left her. I have used numerous astringent lotions, such as solutions of hydr. mur. zinc. vitr. cupr. vitr. aluminis. Decoct. cort. peruv. but without success. I have given the bark both in decoction, in substance with vitriolic acid, and with wine, but without effect. The bals. copaivæ was likewise prescribed, but was so disagreeable to her palate, as to be instantly rejected. To what to attribute the failure of these means, I cannot devise, unless it be from the lotions having never reached the seat of the disease, which is evidently the urethra, all the discharge proceeding from that passage, and not from the vagina. Had I not discovered this to be the case, and known it was in the first instance contagious, although it does not remain to be so, I should have doubted whether or no I had mistaken a case of Fluor Albus for Gonorrhœa.

